# Association between Flexibility, Measured with the Back-Scratch Test, and the Odds of Oxytocin Administration during Labour and Caesarean Section

**DOI:** 10.3390/jcm13175245

**Published:** 2024-09-04

**Authors:** Virginia A. Aparicio, Nuria Marín-Jiménez, Jose Castro-Piñero, Marta Flor-Alemany, Irene Coll-Risco, Laura Baena-García

**Affiliations:** 1 Department of Physiology, Institute of Nutrition and Food Technology, University of Granada, 18003 Granada, Spain; virginiaparicio@ugr.es; 2 Sport and Health University Research Institute (iMUDSmuds), University of Granada, 18007 Granada, Spain; irecollrisco@gmail.com (I.C.-R.); lbaenagarcia@ugr.es (L.B.-G.); 3 Glzartea, Kirola eta Ariketa Fisikoa Ikerkuntza Taldea (GIKAFIT), Society Sports and Exercise Research Group, Department of Physical Education and Sport, Faculty of Education and Sport, Physical Activity and Sport Sciences Section, University of the Basque Country (UPV/EHU), 01006 Vitoria-Gasteiz, Spain; 4 GALENO Research Group, Department of Physical Education, Faculty of Education Sciences, University of Cadiz, 11519 Puerto Real, Spain; jose.castro@uca.es; 5 Instituto de Investigación e Innovación Biomédica de Cádiz (INiBICA), 11009 Cadiz, Spain; 6 Department of Health and Biomedical Sciences, Faculty of Health Sciences, University of Loyola Andalucia, Campus Sevilla, Avda. de las Universidades S/N, 41704 Dos Hermanas, Spain; 7 Department of Nursing, Faculty of Health Sciences, University of Granada, 18071 Granada, Spain; 8 Biosanitary Research Institute, IBS, University of Granada, 18012 Granada, Spain

**Keywords:** pregnant woman, physical fitness, flexibility, oxytocin, labour, obstetric risk

## Abstract

**Objective:** This study explored whether assessing flexibility levels in clinical settings might predict the odds of oxytocin administration and caesarean section to stimulate labour. **Methods:** Pregnant women from the GESTAFIT Project (n = 157), participated in this longitudinal study. Maternal upper-body flexibility was assessed at 16 gestational weeks (g.w.) through the Back-scratch test. Clinical data, including oxytocin administration and type of birth, were registered from obstetric medical records. **Results:** Pregnant women who required oxytocin administration or had caesarean sections showed lower flexibility scores (*p* < 0.05 and *p* < 0.01, respectively). The receiver operating characteristic curve analysis showed that the Back-scratch test was able to detect the need for oxytocin administration ((area under the curve [AUC] = 0.672 (95% confidence interval [CI]: 0.682 (95% CI: 0.59–0.78, *p* = 0.001)). The AUC to establish the ability of flexibility to discriminate between vaginal and caesarean section births was 0.672 (95% CI: 0.60–0.77, *p* = 0.002). A Back-scratch test worse than 4 centimetres was associated with a ~5 times greater increased odds ratio of requiring exogenous oxytocin administration (95% CI: 2.0–11.6, *p* = 0.001) and a ~4 times greater increased odds ratio of having a caesarean section (95% CI: 1.7–10.2, *p* = 0.002). **Conclusions:** These findings suggest that lower flexibility levels at the 16th g.w. discriminates between pregnant women who will require oxytocin and those who will not, and those with a greater risk of a caesarean section than those with a vaginal birth. Pregnant women below the proposed Back-scratch test cut-offs at 16th g.w. might specifically benefit from physical therapies that include flexibility training.

## 1. Introduction

Events related to the labour process have important implications for both the mother and the new-born [1,2,3,4]. Therefore, it is clinically relevant to explore and identify factors that might be associated with a lower risk of common interventions that should be avoided during labour [2,5], such as the exogenous administration of oxytocin [2,4,5,6] and caesarean sections [7].

In this context, we previously observed that greater physical fitness was associated with better labour-related outcomes, such as less need for oxytocin administration to induce or stimulate labour [8] and lower caesarean incidence [9]. Previous studies have shown that muscle stretching exercises during pregnancy are associated with better maternal and neonatal birth outcomes (such as less pelvic pain, greater mobility, better maternal mental state, and lower rate of obstetric complications) [10,11]. In this regard, a randomised clinical trial showed that women who undertook a yoga programme during pregnancy had lower rates of labour induction and caesarean sections [12]. The dimension of physical fitness that tends to increase with this type of intervention is flexibility. However, the role of this variable in relation to induction and/or stimulation of labour and type of birth has not been explored so far. Notwithstanding, most of the tests employed for measuring physical fitness require large spaces, special equipment such as a treadmill, or excessive time for assessment in clinical settings. Consequently, the election of time-efficient measuring tools adapted to health professionals, who usually have less than five minutes of consultation time [13], is mandatory. In this sense, the Back-scratch test, a quick and easy tool for measuring the range of motion that only requires a standard ruler, could be an excellent option. Furthermore, this tool has demonstrated a powerful capacity to predict key health outcomes such as cardiometabolic risk in several populations [14,15], an association with better mental health in healthy women and women with fibromyalgia, and even a role in predicting the risk of fibromyalgia and its severity [16]. Furthermore, within the GESTAtion and FITness (GESTAFIT) project, we have observed that this test is associated with improved maternal and neonatal birth-related outcomes [9]. 

Consequently, the aims of the present study were (i) to identify whether flexibility levels during the early second trimester of pregnancy may predict the need for oxytocin administration to induce or stimulate labour and the type of birth (i.e., vaginal or caesarean section) and (ii) to establish Back-scratch test cut-off points able to improve the accuracy of the need for oxytocin administration and the prognosis of caesarean section as a clinician tool for identifying pregnant women who could benefit from physical therapy programs that include flexibility training.

## 2. Materials and Methods

### 2.1. Study Sample and Design

The detailed procedures and inclusion and exclusion criteria (see Supplementary Table S1) of the GESTAFIT project were previously published [17]. Briefly, pregnant women between 18 and 45 years old with a normal pregnancy course who were able to walk without assistance, write and read properly, and signed an informed consent were eligible for selection. In addition, twin pregnancies, women with acute or terminal illnesses and gestations with foetal pathologies were excluded. This study is a secondary analysis that is part of a larger project in which a concurrent physical exercise program (aerobic plus strength training) was carried out in the intervention group from the 17th gestational week (g.w.) until birth. A total of 384 pregnant women were informed about this study during their 12th g.w. visits to the gynaecologist at the University Hospital. A final number of 159 women were interested in participating and signed an informed consent. Finally, 137 women had complete and valid data in relation to the specific aims of this study. The GESTAFIT project was approved by the Ethics Committee on Clinical Research (CEIC) of Granada, Regional Government of Andalusia, Spain (code: GESFIT-0448-N-15).

### 2.2. Procedures

The first evaluation of this study was carried out during the 16th g.w. The research team was present at all times to provide any explanations or instructions as needed. The pregnant women completed a self-reported questionnaire, anthropometric assessment, and the Back-scratch test. Height and weight were also assessed. Obstetric and gynaecological histories and birth outcomes were collected through the Pregnancy Health Document and digital medical records.

### 2.3. Maternal Sociodemographic and Clinical Data

Sociodemographic (age, number of children and marital, educational, and working status), reproductive history, and clinical (suffering or having suffered specific diseases and drug consumption) data were assessed with a self-reported questionnaire.

### 2.4. Anthropometric Assessment

Height and weight were measured using a stadiometer (Seca 22, Hamburg, Germany) and a scale (InBody R20; Biospace, Seoul, Republic of Korea), respectively. The body mass index was calculated as weight (kg)/height (m^2^).

### 2.5. Pregnancy Health Document: Obstetric during Pregnancy and Pregnancy History

The “Pregnancy Health Document” is given to all pregnant women by the Andalusian regional government, and it contains obstetric and medical data recorded during the whole pregnancy. In this way, information about previous pregnancies and births and gynaecological antecedents were obtained. Gestational age was calculated by the date of last menstruation corrected for cycles of 28 days. 

### 2.6. Labour Outcomes

All data related to the type of birth (vaginal or caesarean), gestational week at birth, use of epidural analgesia, offspring sex, neonatal weight, and the Apgar test were obtained from perinatal obstetric records (partogram) from the hospital after birth. 

#### Oxytocin Administration before or during Labour

Information about the use of oxytocin was collected from the partogram. In this document, midwives usually record whether oxytocin is administered or not, but the dose and administration time are not frequently collected, so these data were not assessed in the present study. Moreover, we considered that oxytocin was administered both by induction of labour and uterine stimulation, but we did not consider the administration of this drug during placenta birth. 

### 2.7. The Back-Scratch Test

**Figure 1:**
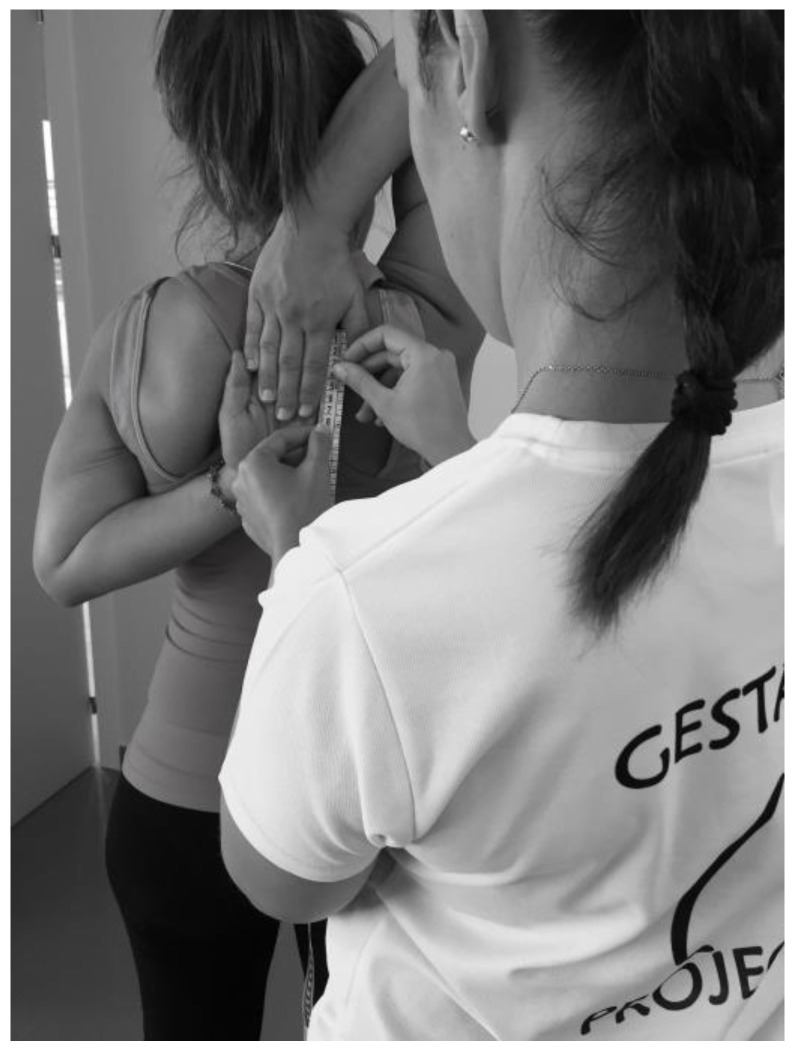
“Back-scratch” test assessment.

The Back-scratch test [18] was used to assess upper-body flexibility (Figure 1). The test consists of measuring the overall shoulder range of motion by measuring the distance between (or overlap of) the middle fingers as they come together behind the back. This test was performed twice with both hands, alternatively; the final score in centimetres (cm) was calculated as the mean value of the best attempts for both arms.

### 2.8. Statistical Analyses

Descriptive statistics were summarized as mean (standard deviation) for quantitative variables and frequency (%) for categorical variables.

**Figure 2:**
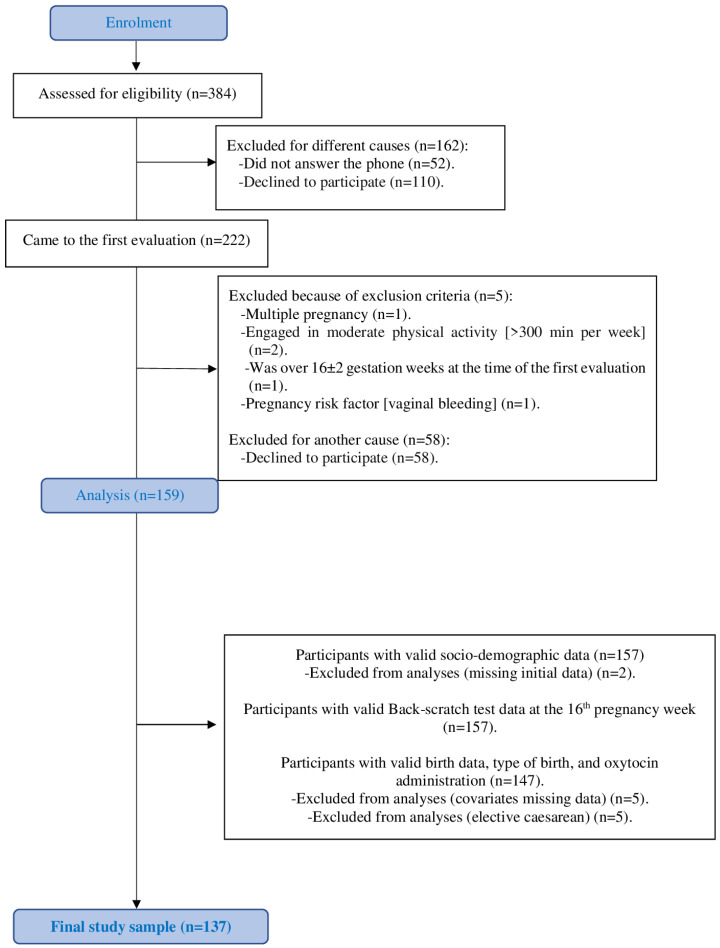
Flow diagram of the study participants.

The comparisons of the Back-scratch test between pregnant women with and without oxytocin administration and with and without caesarean section were performed by the T-student test and one-way analysis of covariance (ANCOVA) after adjustment for maternal age and weight, parity, the exercise intervention, epidural analgesia, and birth place. Planned caesarean sections were excluded from the analyses (n = 5, Figure 2). Furthermore, standardized effect size statistics were estimated for these comparisons through Cohen’s *d* and its exact confidence interval (CI). The effect size was interpreted as small (~0.2), medium (~0.5) or large (~0.8 or greater).

The Back-scratch test thresholds that best prognosticated the subjects as having vs. not having oxytocin administration and as having vs. not having a caesarean section were determined by using receiver operating characteristic (ROC) curve analysis [19].

The ROC curve is a plot of all the sensitivity/specificity pairs resulting from varying the decision threshold [19]. To identify the best threshold, the distance between the perfect test and each sensitivity and 1-specificity pair was calculated, and then, the pair closest to 1 was chosen. We also calculated the area under the curve (AUC) and the 95% CI. The AUC represents the ability of the Back-scratch test to correctly classify subjects as having vs. not having oxytocin administration and having vs. not having a caesarean section as having vs. not having oxytocin administration. The values of AUC range between 1 (perfect test) and 0.5 (worthless test). 

Binary logistic regression was used to further study the relationship among the Back-scratch test-derived cut-offs, oxytocin administration, and the presence/absence of caesarean section. Maternal age and weight, parity, maternal, exercise intervention, epidural analgesia, and birth place were also additionally included as covariates to test their potential confounder effects on upper-body flexibility and the risk of oxytocin administration and caesarean section. 

All the analyses were performed using the Statistical Package for Social Sciences (IBM SPSS Statistics for Windows, version 26.0; Armonk, NY, USA), and the level of significance was set at *p* < 0.05.

## 3. Results

Of the 159 women who met the eligibility criteria and completed the first assessment, 157 women had complete and valid sociodemographic data. However, data on birth type and oxytocin administration were missing for 15 participants, and five pregnant women were excluded from the analyses because they had elective caesarean sections (see Figure 2).

The sociodemographic and clinical characteristics of the study participants are shown in Table 1. The final sample size was composed of 137 Caucasian pregnant women (aged 32.9 ± 4.6 years old, 66.7 ± 11.9 kg of mean weight at the 16th g.w.). Most of the participants lived with their partners (97%), had University degrees (57%), and worked full time. Approximately 61% of the sample were nulliparous, and 23% had a caesarean section. More than half of the caesarean sections (55%) were due to failure of labour progression (prolonged labour). Births took place around 39.6 ± 1.3 g.w., with a mean neonate body weight of 3.3 ± 0.5 kg. The mean value of the Back-scratch test was 4.1 ± 6 cm.

Differences in the Back-scratch test of the pregnant women at the 16th g.w. by oxytocin administration and type of birth are shown in Table 2. The mean scores in the Back-scratch test were +1.8 cm in women who needed oxytocin administration compared with +5.4 cm in women who did not require its administration (*p* = 0.001 for the unadjusted model and *p* = 0.004 for the adjusted model, Cohen’s *d* = 0.59, 95% CI: 0.2–0.95). The mean cm values in the Back-scratch test were +1.6 cm in women who had caesarean sections compared with +5.0 cm in women who had vaginal births (*p* = 0.004 for the unadjusted model and *p* = 0.017 for the adjusted model, Cohen’s *d* = 0.55, 95% CI: 0.2–0.9).

**Figure 3:**
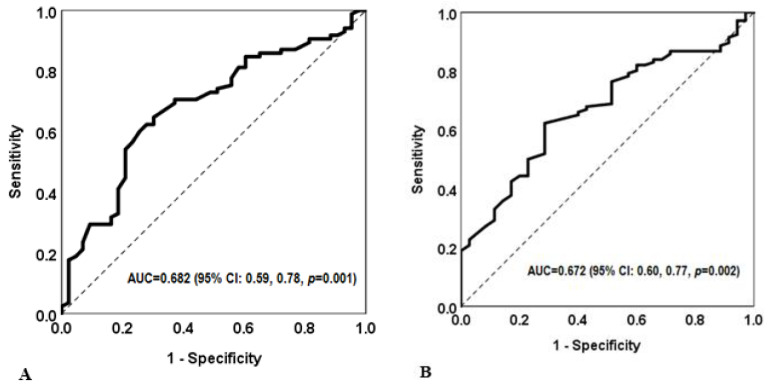
(**A**) Capacity of the Back-scratch test to discriminate the need (or not) for oxytocin administration before or during labour. (**B**) Capacity of the Back-scratch test to discriminate between vaginal birth and caesarean section.

Figure 3 shows the capacity of the Back-scratch test to discriminate between the need for oxytocin administration before or during labour (Figure 3A) and presence/absence of caesarean section (Figure 3B). The AUC to establish the ability of the Back-scratch test to detect the need for oxytocin administration was 0.682 (95% CI: 0.59, 0.78, *p* = 0.001). The AUC to establish the ability of the Back-scratch test to detect the odds of caesarean section was 0.672 (95% CI: 0.60, 0.77, *p* = 0.002).

The thresholds derived from the ROC analysis for the need for oxytocin administration and the presence/absence of caesarean section are shown in Table 3. The optimal cut-off to discriminate the need for oxytocin administration was +3.6 cm (OR = 4.2; 95% CI: 1.9–9.3 for the unadjusted model, and OR: 4.8; 95% CI: 2.0–11.6.7 for the adjusted model). The cut-off points, ORs, and 95% CIs of the Back-scratch test to identify caesarean presence were tested in an unadjusted model after adjusting for maternal age and weight, parity, exercise intervention, epidural analgesia, and birth place. The optimal cut-off point to discriminate between the presence and absence of a caesarean section was +4.1 cm (OR: 4.1; 95% CI: 1.8–9.5 for the unadjusted model, and OR: 4.2; 95% CI: 1.7–10.2 for the model adjusted for the abovementioned potential confounders).

## 4. Discussion

The main findings of the present study indicate that lower flexibility levels during the early second trimester of pregnancy may be indicators of the need for oxytocin administration before or during labour and caesarean section.

At the 16th g.w., a Back-scratch test score < 3.6 cm was associated with a ~5 times greater increased odds ratio for requiring exogenous oxytocin administration to induce or stimulate labour. A Back-scratch test score < 4.1 cm was associated with a ~4 times greater increased odds ratio for having a caesarean section. The proposed cut-offs provide useful information for clinical settings that can be used to recommend a potential tailored prescription of flexibility training programs during pregnancy.

Within the GESTAFIT project, our group previously showed that maternal physical fitness is a key factor related to maternofoetal health and birth outcomes [8,9]. The present results support our previous findings and highlight the importance of implementing physical fitness testing as a complementary tool for the screening of healthy pregnancies. Therefore, considering that the Back-scratch test is efficient in terms of time and equipment, we propose its use as a powerful test to be implemented in routine clinical practice.

Since women who required oxytocin administration showed lower flexibility during the early second trimester of pregnancy, this physical fitness component might be key in preventing the need for this intervention. According to the Spanish Ministry of Health, Social Services, and Equality, the prevalence of the use of exogenous oxytocin during spontaneous labour in Spanish public hospitals is 53%, which is much higher than the recommended standards of 5–10% [20]. In the present study, 34% of women were provided with this hormone during labour, which represents almost four times the recommendations. Synthetic oxytocin is extensively employed as a method to induce labour [21,22] and a treatment for dystocia of uterine dynamics [22]. However, its use has been related to increased risk of uterine hyperactivity, alterations in the foetal heart rate, and postpartum haemorrhage [7]. In addition, other studies have associated the use of oxytocin with sucking problems and early cessation of breastfeeding [4], among other neonatal complications [6].

It was previously shown that maternal flexibility was associated with a lower incidence of caesarean sections [8], and modalities of exercise widely recommended during pregnancy that prioritize flexibility training, such as yoga, have been related to higher rates of vaginal births [12]. To note, caesarean sections are clearly associated with greater postpartum complications for the mother and new-born [1,2,3]. In our study sample, 25% of the births were caesarean sections, a much higher rate than the one recommended by the World Health Organization, which establishes that rates above 15% do not reduce maternal and neonatal morbidity and mortality [23]. It should also be taken into account that in Spanish private hospitals, the caesarean ratio is higher than in public hospitals, which we considered by including the place of birth as a potential confounder. 

Several mechanisms might partially explain the role of flexibility in the type of delivery and the need for oxytocin administration. First, overall bodily flexibility levels may be related to the status of the connective tissue (i.e., ligaments) during pregnancy, which may present greater ligament laxity, which is necessary for the correct maintenance of pregnancy and labour progression. Second, pregnant women with greater flexibility might also present greater serum relaxin concentrations [23], which are also naturally increased during pregnancy [24]. Relaxin is a key hormone during pregnancy that also powerfully increases ligament laxity [25] and, consequently, body flexibility. Third, relaxin also provides vasodilator effects [26], which promote enhanced blood flow to the foetus and reduce potential alterations in foetal well-being. Moreover, since relaxin has endothelium-dependent vasodilation effects in the uterine artery [26], it seems feasible that the uteroplacental flow was more efficient during labour in women with greater relaxin concentrations—and probably also higher body flexibility. In this line, in a previous study [9], we found that greater maternal flexibility at the 16th gestational week was associated with a more alkaline pH, higher PO^2^, higher arterial oxygen saturation, and lower PCO^2^ in the arterial umbilical cord blood. Fourth, it seems that high levels of relaxin might also have a determinant role in the appearance of uterine contractions [27,28]. Finally, although more studies are needed to confirm this hypothesis, it is possible that women with better cardiometabolic status—which has been highly associated with the Back-scratch test scores in several populations [14,15]—showed greater cardiorespiratory fitness [29] and, therefore, experienced less fatigue during labour. Less fatigue promotes better uterine dynamic [30], and fatigue is also one of the main clinical reasons for providing this hormone during labour [23].

This study has several clinical implications to highlight. The high capacity of the Back-scratch test to establish the odds of the need for oxytocin administration and caesarean section, and the fact that it is a very accessible tool, reinforces that it should be included as a new complementary pregnancy screening tool. Particularly, the Back-scratch test has great potential in a clinical setting for the following reasons: (i) a measuring tape or a ruler is all the equipment needed to perform this test, so it is extremely cheap; (ii) the procedures for this test are simple and do not require any particular training; (iii) typically, physical fitness tests require larger spaces, while the Back-scratch test can be performed in any room without any special requirement; and (iv) this test is time-efficient, requiring just one minute, which is a fundamental issue for clinicians who are usually under time constraints.

As our intention is the prompt detection of these common obstetric risks, we encourage clinicians to assess this test around the 16th g.w. in order to initiate prevention strategies focused on flexibility early. From the GESTAFIT project team, we highly recommend those preventive interventions focused on physical exercise [8,9,31], as it exerts strong positive effects on birth-related outcomes such as the prevalence of caesarean sections, gestational age, length of labour stages, birth weight, Apgar test scores, and umbilical cord blood gases, among others [8,9,31]. We also recommend incorporating flexibility training in pregnant women below the cut-offs. Future studies are warranted to check the influence of specific flexibility programs (e.g., yoga, stretching) on women below the proposed cut-offs in order to explore their potential positive influences on birth outcomes through flexibility gains.

Some limitations must be highlighted. The study sample was relatively small, and we have missing data for different reasons, so studies with larger sample sizes are needed to establish more robust cut-off points. Moreover, because of the relatively small sample size, we could not further establish age-specific cut-off points (e.g., for women aged more or less than 30 years old).

This study also has several strengths to note. As far as we know, this is the first study establishing simple physical flexibility test cut-off points for the monitoring of pregnancies in clinical settings. Further, this test might also provide a powerful preventive tool for clinicians. Moreover, we confirmed the potential of the Back-scratch test after the adjustment for relevant potential confounders that could affect flexibility or the risk of caesarean section and complicated births, such as maternal age and weight, parity, birth place or the use of epidural analgesia.

## 5. Conclusions

Overall, women who needed oxytocin administration or suffered a caesarean section showed lower flexibility levels. The early identification of pregnant women who fail to meet the suggested standards in the Back-scratch test can assist in better pregnancy monitoring and might help to identify relevant birth-related complications easily, quickly, and cheaply and then initiate preventive strategies (for instance, focused on improving flexibility levels within their exercise program).

The Back-scratch test should be proposed as a discriminative tool for predicting the need for oxytocin administration during labour and the odds of caesarean section. A Back-scratch test score <3.6 cm was associated with a ~5 times greater increased odds ratio of requiring exogenous oxytocin administration to induce or stimulate labour. A Back-scratch test score <4.1 cm was associated with a ~4 times greater increased odds ratio of having a caesarean section. Therefore, optimal flexibility levels during pregnancy might prevent these labour-related complications.

## Figures and Tables

**Table 1:** Sociodemographic and clinical characteristics of the study participants (n = 137).

Maternal Outcome	Mean (SD)
Age, years	32.9 (4.6)
Body mass index at 16th gestational week, kg/m^2^	24.9 (4.1)
Weight at 16th gestational week, kg	66.7 (11.9)
	n (%)
Living with a partner, yes	133 (97.1)
Educational status	
Primary or highschool	33 (24.1)
Specialized training	26 (18.9)
University degree	78 (56.9)
Working status	
Homework/unemployed	41 (29.9)
Partial-time employed/student	37 (27)
Full-time employed	59 (43.1)
Oxytocin administration to induce or stimulate labour	44 (32.1)
Epidural analgesia, yes	94 (72.9)
Type of birth	
Vaginal	106 (77.4)
Non-planned caesarean section	31 (22.6)
Reason of caesarean section	n (%)
Risk of loss of foetal well-being	9 (29)
Failed induction	2 (6.5)
Failure to progress	17 (54.8)
Suspected Cephalopelvic Disproportion	3 (9.7)
Birth place	
Public hospital	131 (95.6)
Private hospital	5 (3.6)
Home	1 (0.7)
Parity	
Nulliparous	84 (61.3)
Multiparous	53 (38.7)
Back-scratch test	Mean (SD)
	4.1 (6.2)
16th gestational week	Median
	4.7
Neonatal outcome	
Sex (female, n (%))	68 (50.7)
Gestational age at birth, weeks	39.6 (1.3)
Birth weight, grams	3314 (482.8)
Apgar Test 1 min	8.6 (1.0)
Apgar Test 5 min	9.6 (0.7)

**Table 2:** Differences in the Back-scratch test of the pregnant women at the 16th gestational week by oxytocin administration and type of birth.

Oxytocin Was Not Administered (n = 93)	Oxytocin Was Administered (n = 44)	*p*	p *	Effect Size *d*-Cohen (95% CI)
5.40 (0.68)	1.76 (0.89)	0.001	0.004	0.59 (0.23, 0.95)
Vaginal Birth (n = 106)	Caesarean Section (n = 31)	*p*	p *	Effect Size *d*-Cohen (95% CI)
5.04 (0.63)	1.61 (0.89)	0.004	0.017	0.55 (0.21, 0.89)

Values are shown as mean (standard error of the mean). CI, confidence interval. * Model adjusted for maternal age, parity, maternal weight, exercise intervention, epidural analgesia, and birth place.

**Table 3:** Binary logistic regression statistics testing the predictive capacity of the Back-scratch test thresholds derived from the receiver operating characteristics curve analysis for the need for oxytocin administration before or during labour and the presence/absence of caesarean section.

	Low Back-Scratch Test (Based on the Cut-Off)						
	Cut-Off Point (cm)	Unadjusted Model			Adjusted Model *		
		OR	95% CI	*p*	OR	95% CI	*p*
Oxytocin administration	<3.6	4.23	1.92–9.31	<0.001	4.79	1.97–11.6	0.001
Caesarean section	<4.1	4.13	1.80–9.50	0.001	4.15	1.70–10.2	0.002

High Back-scratch test was used as reference; OR, odds ratio; CI, confidence interval; * Model adjusted for maternal age, parity, maternal weight, exercise intervention, epidural analgesia, and birth place.

## Data Availability

The data that support the findings of this study are available from the corresponding author, M.F.A., upon reasonable request.

## Supplementary Materials

The following supporting information can be downloaded at https://www.mdpi.com/article/10.3390/jcm13175245/s1, Table S1: Study inclusion and exclusion criteria.
