# Supplementary material for: Association between Flexibility, Measured with the Back-Scratch Test, and the Odds of Oxytocin Administration during Labour and Caesarean Section
Source: J Clin Med. 2024 Sep 4;13(17):5245. doi: 10.3390/jcm13175245 (PMC11396226; doi:10.3390/jcm13175245)
Supplement: Supplementary file 1 [file jcm-13-05245-s001.zip › jcm-3136341-supplementary.pdf]

Table S1. Study inclusion and exclusion criteria.

Inclusion criteria
- Pregnant women aged 25-40 years old with a normal pregnancy course.
- Answering "no" to all questions on the PARmed-X for pregnancy.
- Being able to walk without assistance.
- Being able to read and write properly.
- Informed consent: Being capable and willing to provide written consent.
*In addition, specific inclusion criteria for data analysis are: pregnancy with single foetus, spontaneous or instrumental vaginal birth, and caesarean without maternofoetal pathology (or any other indication involving maternofoetal risk, such as disproportion, failed induction, no foetal progression or non-cephalic presentation).
Exclusion criteria
- Acute or terminal illness.
- Malnutrition (previous diagnoses related to the follow-up of non-nutrient balanced diets with potential micro- and/or macronutrient deficits).
- Inability to conduct tests for assessing physical fitness or exercise during pregnancy.
- Underweight (less than 18.5 on Body Mass index).
- Pregnancy risk factors (such as hypertension, type 2 diabetes, etc.).
- Multiple pregnancies.
- Chromosopathy or foetal malformations.
- Intrauterine growth restriction (Estimated Fetal Weight < 10th%).
- Foetal death.
- Upper or lower extremity fracture in the past 3 months.
- Presence of neuromuscular disease or drugs affecting neuromuscular function.
- Doing more than 300 minutes of at least moderate physical activity per week.
- Being registered in another exercise program.
- Unwillingness either to complete the study requirements or to be randomized into the control or intervention group.
